# *Candidozyma auris* in The Netherlands: No Evidence of Nosocomial Transmission Supported by Effective Infection Control Policies

**DOI:** 10.1007/s11046-025-01024-7

**Published:** 2026-01-10

**Authors:** Chiara C. de Groot, Bram Spruijtenburg, Juliëtte A. Severin, Karin van Dijk, Jochem B. Buil, Paul E. Verweij, Auke W. de Jong, Eelco F. J. Meijer

**Affiliations:** 1 Centre for Infectious Disease Control, National Institute for Public Health and the Environment (RIVM), Bilthoven, The Netherlands; 2 Department of Medical Microbiology, Radboud University Medical Center, Nijmegen, The Netherlands; 3 Radboudumc-CWZ Center of Expertise for Mycology, Nijmegen, The Netherlands; 4 Department of Medical Microbiology and Immunology, Canisius-Wilhelmina Hospital (CWZ)/Dicoon, Nijmegen, The Netherlands; 5 Department of Medical Microbiology and Infectious Diseases, Erasmus MC, Rotterdam, The Netherlands; 6 Department of Medical Microbiology and Infection Prevention, Amsterdam University Medical Center, Amsterdam, The Netherlands

**Keywords:** Candidozyma auris, Outbreak, Infection prevention, Surveillance, Whole genome sequencing, Antifungal resistance

## Abstract

The yeast *Candidozyma auris* has emerged globally as a major threat to public health. Outbreaks are frequently reported and difficult to control. In the Netherlands, *C. auris* is rarely detected although national surveillance has been set up. Here, we present all Dutch *C. auris* cases reported from March 2018 until April 2025. Antifungal susceptibility testing (AFST) using broth microdilution and whole genome sequencing (WGS) were conducted to evaluate antifungal resistance and genetic relatedness. A total of 26 cases of *C. auris* infection or colonization were reported across 22 different medical institutions in the Netherlands. Most patients had a history of travel to countries with prior reports of *C. auris* and were hospitalized in foreign medical centers. All patients were admitted in isolation, and all but one remained in isolation for the duration of their hospitalization. WGS showed isolates belonged to clade I or III. Analysis of travel history, contact tracing and WGS data showed no evidence of nosocomial transmission. All isolates were non-wild type to fluconazole with many harboring corresponding mutations in *ERG11*. One isolate was non-wild type to 5FC and another one to echinocandins including rezafungin. The latter harbored a *FKS1*^*F635Y*^ mutation and was imported from Greece. To conclude, *C. auris* cases are steadily increasing in the Netherlands. Nonetheless, until now cases seem solely imported from abroad with no evidence for nosocomial transmission. This can be attributed to effective infection prevention and control policies. The *C. auris* isolates were all non-wild type for fluconazole and a single isolate was non-wild type for echinocandins.

**Supplementary Information:**

The online version contains supplementary material available at 10.1007/s11046-025-01024-7.

## Introduction

There is a global rise in colonization and infections by *Candidozyma auris* (formerly known as *Candida auris*) [1]. This fungal pathogen is a serious health care threat recognized by the World Health Organization (WHO) that has included *C. auris* in the critical priority group of the fungal priority pathogen list [2]. *C. auris* can cause invasive candidiasis, with candidemia causing an overall mortality of over 40% in Europe [3]. Risk factors for candidemia include the insertion of catheters and other medical devices, prior antibiotic treatment, extended stay in an intensive care unit (ICU) and prior surgery [4]. Specifically for *C. auris*, intramural colonization is a risk factor for invasive disease and patients with foreign bodies in situ, i.e. central venous lines and/or intubated, with an inter-hospital transfer are most at risk for colonization [5, 6]. Among the unique characteristics of this yeast is its high outbreak potential, causing numerous large outbreaks globally [7–9]. Outbreak control and subsequent eradication from the hospital can be challenging. Colonized patients can spread *C. auris* to medical devices as well as the innate hospital environment, where it can remain viable for weeks [10, 11]. Also, patients can remain colonized for extended periods of time and decolonization strategies have been proven ineffective [4, 10].

Based on whole genome sequencing (WGS) analysis *C. auris* can be grouped into six geographically distinct clades: Clade I (South Asia), Clade II (East Asia), Clade III (Africa), Clade IV (South America), Clade V (Iran), and Clade VI (Indomalayan) [12–14]. Differences among clades vary from tens of thousands to hundreds of thousands of single nucleotide polymorphisms (SNPs) [14], contrasting the intra-clade diversity that is extremely low (< 500 SNPs) [15]. Moreover, these clades differ in their antifungal resistance, which is overall high when compared to other *Candida* species [4]. A recent study analyzing data from 67 studies, covering over 4,000 cases, showed that *C. auris* is often resistant to fluconazole (91%) with lower resistance rates to amphotericin B and echinocandins, although there are large differences among clades and outbreaks [16, 17]. Notably, resistance mechanisms and mutations differ among clades, highlighting the critical importance of using validated antifungal susceptibility testing (AFST) methodologies. Clade II is overall susceptible, whereas clades I and III are nearly always resistant to fluconazole [15]. Additionally, clade I has the highest rates of multidrug resistance with even pan-resistant strains reported [18].

Infection prevention and control measures (IPC) are important to prevent and control the spread of *C. auris* and other multidrug-resistant organisms (MDROs) [19, 20]. The Netherlands is renowned for having relatively low antimicrobial resistance rates compared to other European countries [21], largely attributed to its effective IPC measures that are described in national guidelines [22]. As of October 2024, *C. auris* has been included in the national MDRO guideline by the Dutch Collaborative Partnership for Infection Prevention Guidelines (SRI) [23]. Originally, the MDRO guideline was designed for resistant bacteria but has now incorporated *C. auris*, using protocols already established for resistant bacteria. Notably, a survey conducted prior to the inclusion of *C. auris* in the MDRO guidelines revealed that in many Dutch hospitals screening and outbreak protocols for this microorganism were lacking [24]. In Europe, the prevalence of cases and outbreaks varies highly between countries, with some countries like Belgium and the Scandinavian countries that reported only a few cases to date, while others like the United Kingdom, Italy, Greece and Spain already reported large outbreaks [9, 25]. Regarding the presence of *C. auris* in the Netherlands, only a few cases have been described in literature [26–28]. No recent data has been reported on the occurrence of *C. auris* in the Netherlands, leaving the current epidemiological status unclear. Therefore, in this article we provide an overview of all *C. auris* cases in the Netherlands that were reported to the RIVM/Radboudumc-CWZ National Reference Laboratory for Invasive Mycoses. This includes a description of the epidemiology, implemented infection prevention measures, genomic relatedness and an overview of the antifungal resistance patterns with underlying mechanisms.

## Method

### Isolate Collection

In the Netherlands, all laboratories are requested to submit the first *C. auris* isolate from each confirmed colonized or infected patient to the RIVM/Radboudumc-CWZ National Reference Laboratory for Invasive Mycoses on a voluntary base, as there is no mandatory reporting. This study includes all *C. auris* isolates submitted to the reference laboratory from March 2018—when the first *C. auris* case was detected in the Netherlands – until April 2025. A total of 26 cases with *C. auris* were included in the study.

Collection methods varied between institutions, isolates from both screening and diagnostic cultures were included. Two isolates from the same patient were considered distinct cases only if the patient was declared *C. auris* free in the interim. According to the MDRO guideline by the SRI, a patient is considered *C. auris* free after two negative cultures obtained one year following the last positive culture [23]. In this study, no patients experienced multiple cases; all isolates originated from different individuals.

All medical centers with a confirmed *C. auris* case were contacted to collect information on patient characteristics and implemented infection control measures. To describe the epidemiology, data on age, sex, the body sites that tested positive for *C. auris*, travel history, and residency in a long-term care facility or asylum seeker center were requested. Additionally, to assess the risk of transmission, information was collected on whether patients were placed in isolation during hospitalization, whether contact tracing was conducted, and whether any transmission events were identified.

No ethical approval was required according to the Dutch Framework for the assessment of medical-scientific research not subject to the WMO by the Ministry of Health, Welfare and Sport. The COREON code of conduct for health research has been followed.

### Species Identification and AFST

Species identification was confirmed by Matrix Assisted Laser Desorption Ionization-Time of Flight Mass Spectrometry (MALDI-TOF MS) (Bruker, Bremen, Germany). AFST was performed following the European Committee on Antimicrobial Susceptibility Testing (EUCAST) E.Def 7.4 broth microdilution method [29]. Each isolate was tested for susceptibility to the following antifungals: amphotericin B (AMB; Sigma, St. Louis, MO, USA), flucytosine (5FC; Sigma, St. Louis, MO, USA), fluconazole (FLC; Pfizer b.v., Capelle aan den IJssel, Netherlands), itraconazole (ITC; Sigma, St. Louis, MO, USA), voriconazole (VRC; Sigma, St. Louis, MO, USA), posaconazole (POS; Sigma, St. Louis, MO, USA), isavuconazole (ISA; Merck, Darmstadt, Germany), anidulafungin (AFG; Pfizer b.v. Capelle aan den IJssel, Netherlands), micafungin (MFG; Astellas Pharma, Leiden, Netherlands), rezafungin (RZF; Mundipharma, Frankfurt, Germany), ibrexafungerp (IBX; medchemexpres, Princeton, NJ, USA), and manogepix (MGX; medchemexpres, Princeton, NJ, USA). Minimum inhibitory concentrations (MICs) were read after 24 h with a spectrophotometer. Isolates were interpreted as wild type or non-wild type according to the established EUCAST tentative epidemiological cutoff values of 0.125 mg/L for RZF, 0.25 mg/L for AFG, 0.25 mg/L for MFG, 2 mg/L for AMB, and 0.5 mg/L for 5FC [30]. Additionally, the epidemiological cutoff values at 97.5% determined by Arendrup et al. of 1 mg/L for ITC, 4 mg/L for VRC, 0.25 mg/L for POS and 0.25 mg/L for ISA were used [31]. For FLC the EUCAST non-species related breakpoint for *Candida* of 4 mg/L was used [32].

### Genotypic Investigation

All isolates were grown on Sabouraud dextrose agar (SDA, Oxoid, Hampshire, United Kingdom) for 48 h at 35 °C. For short tandem repeat (STR) genotyping, DNA was extracted using the MightyPrep Reagent for DNA (Takara Bio Inc., Shiga, Japan) according to manufacturer's instructions. Next, multiplex PCR, amplifying 12 microsatellite markers was conducted, copy numbers were called and were used to determine the genetic relatedness between isolates as previously described [33]. Clade assignment was done by comparison with control strains [33].

For WGS analysis, DNA isolation was performed on the Maxwell RSC 48 platform (Promega, Madison WI, USA) with a pretreatment step of beadbeating and enzymatic lysis. Cultured *C. auris* was suspended in TE with 0.1 mm zirconium beads (BioSpec, Bartlesville, UK) for 2 min at 3800 strokes/min in the Mini-Beadbeater 24 (BioSpec, Bartlesville, UK) followed by enzymatic lysis with 500 U/ml lyticase from Arthrobacter luteus (Sigma-Aldrich, Saint Louis, MO, USA) for 15 min at 37 °C. Genomic DNA purification was then performed using the Maxwell RSC Cultured Cells DNA kit (Promega, Madison WI, USA). Library preparation was performed using the Illumina DNA Prep kit (Illumina, San Diego, CA, USA) and subsequent libraries were sequenced on the Illumina NextSeq 2000 platform. Isolate NL-02 was already sequenced, therefore the publicly available WGS data under BioProject ID: PRJNA560710 was used [28].

Raw read data were aligned to the *C. auris* B11220 (GCA_003013715.2) reference genome using BWA-MEM v0.7.19. Resulting BAM files were filtered, and variant calling was performed with a validated pipeline as described in detail [34]. Three control isolates of each clade were extracted from the National Center for Biotechnology Information (NCBI) database and included in the single nucleotide polymorphism (SNP) analysis (Table S1). Resistance associated genes *ERG11* (OL742093.1) and *FKS1* (OQ632644.1) were located in the *C. auris* B11220 reference genome and isolates were visually inspected for missense mutations. Raw WGS data generated in the current study have been deposited to the NCBI Sequence Read Archive (SRA) database under BioProject PRJNA1272057.

## Results

### Epidemiology

**Fig. 1:**
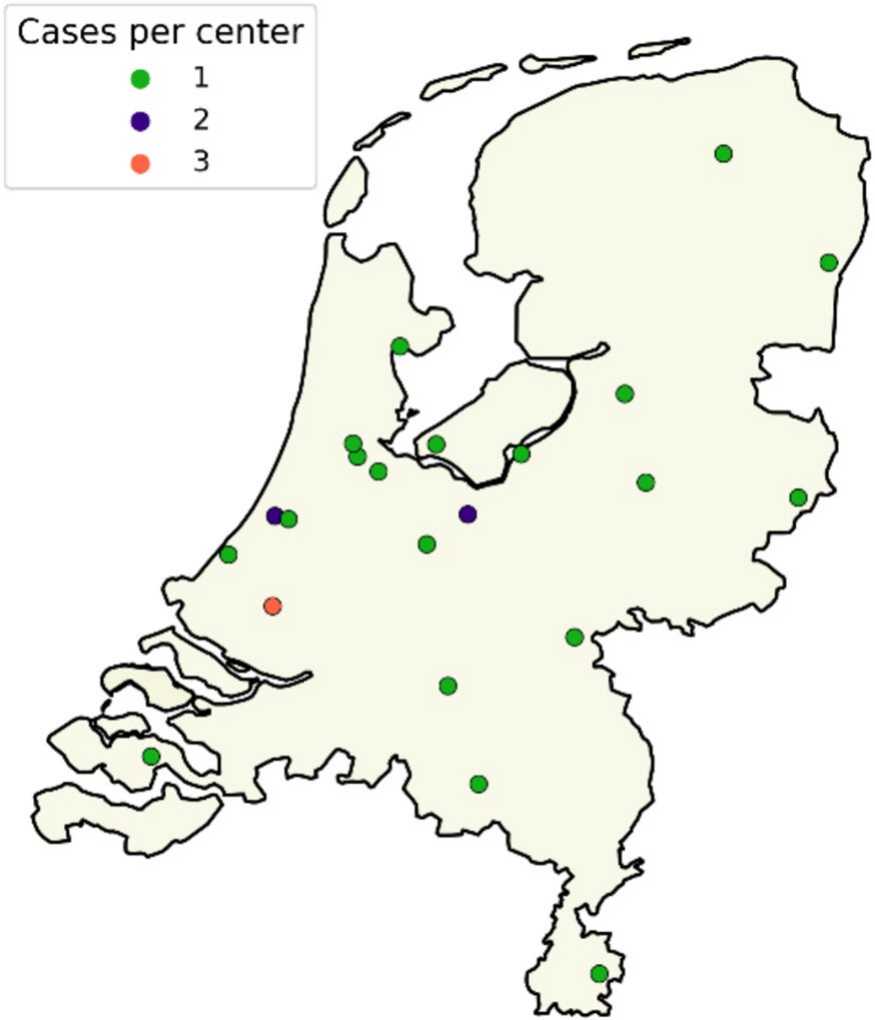
Distribution of 26 *C. auris* cases across the Netherlands. Number of cases per center are indicated by color: green = 1 case, blue = 2 cases, red = 3 cases

**Fig. 2:**
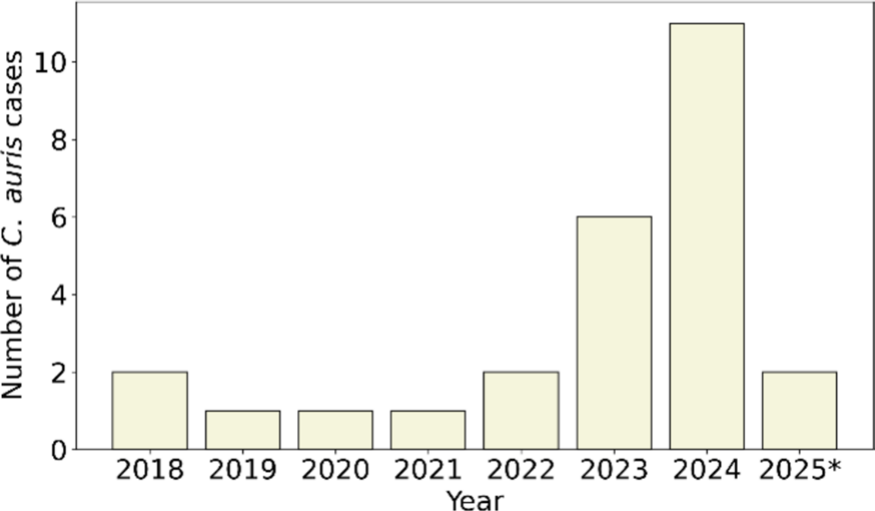
Number of *C. auris* cases per year in the Netherlands. * For 2025 only cases detected until April are shown

The first case of *C. auris* in the Netherlands was detected in March 2018 [28]. Since then, the total number of cases reported to the RIVM/Radboudumc-CWZ National Reference Laboratory for Invasive Mycoses has risen to 26 as of April 2025. These cases were reported by 22 different medical centers and no potential nosocomial transmission was reported. The cases were geographically dispersed across the Netherlands (Fig. 1). An upward trend in the number of reported cases has been observed over the years (Fig. 2). Between 2018 and 2022, only one to two cases were reported annually. The incidence increased in 2023 and 2024 with six and eleven cases respectively. In the first three months of 2025, two cases were reported.

Patient characteristics and relevant medical history were collected from all centers and summarized in Table 1. The *C. auris* patients were distributed across all age groups with a mean age of 54 years, seven patients were female and nineteen male. Information on travel history was available for 24 patients, all of whom had recently traveled abroad. Frequently visited countries included Greece (n = 7), Türkiye (n = 4), South-Africa (n = 4), Tanzania (n = 3), India (n = 2), and Ukraine (n = 2). With the exception of one patient, all had recently been admitted to a foreign healthcare facility. None of the patients were reported to have resided in a Dutch long-term healthcare facility prior to testing positive for *C. auris*. One patient (NL-13) was reported to have been residing in a Dutch asylum seeker center at the time of diagnosis, while two others (NL-08 and NL-24) had been injured during the war in Ukraine. Most common positive sampling areas were axilla and/or groin (n = 15), urine (n = 8), wound (n = 7) and rectum (n = 7). Importantly, two patients had an invasive infection as they had positive blood cultures. The remaining isolates were collected from non-sterile sites and regarded as colonization.

**Table 1:** Epidemiology of Dutch *C. auris* cases

Case	Clade	Date	Isolation sites	Travel history	Admission to a foreign hospital	Treated in isolation	Contact tracing
NL-01	I	2018	Central venous catheter, Groin	India	India	Yes: S	No
NL-02	I	2018	Urine	India	India	Yes: S	No
NL-03	I	2019	Wound	Tanzania & Kenya	Kenya	Yes: I	Yes
NL-04	III	2020	Throat, Nose, Ear	South Africa	South Africa	Partially: S & I	Yes
NL-05	I	2021	Urine, Sputum	Türkiye	Türkiye	Yes: I	No
NL-06	III	2022	Anus, Mediastinum	South Africa	South Africa	Yes: S & I	No
NL-07	I	2022	Rectum	Tanzania	Tanzania	N/A	No
NL-08	III	2023	Axilla	Ukraine	Ukraine	Yes: I	No
NL-09	I	2023	Smear˚	NA	NA	Yes: I	No
NL-10	I	2023	Groin	Greece	Greece	Yes: I	Yes
NL-11	I	2023	Superficial wound, Axilla/Groin	Greece	Greece	Yes: S & I	Yes
NL-12	III	2023	Rectum, Urine	South Africa	South Africa	Yes: I	No
NL-13	I	2023	Ear	Ethiopia, Sudan, Türkiye & Greece	No	N/A*	Yes
NL-14	I	2024	Groin	Tanzania	Tanzania	Yes: S & I	No
NL-15	I	2024	Urine, Cornea, Wound, Axilla, Groin, Nose, Catheter	Greece	Greece	Yes: I	No
NL-16	III	2024	Bloodˣ, Throat, Central venous catheter, Urine	South Africa	South Africa	Yes: I	No
NL-17	I	2024	Rectum, Axilla, Groin, Wound	Bosnia	Bosnia	Yes: S & I	Yes
NL-18	I	2024	Axilla, Groin, Throat, Rectum, Sputum	Greece	Greece	Yes: I	No
NL-19	III	2024	Axilla, Groin	Spain	Spain	Yes: I	No
NL-20	I	2024	Groin	Greece	Greece	Yes: I	No
NL-21	I	2024	Axilla/Groin, Rectum	Türkiye	Türkiye	Yes: I	Yes
NL-22	I	2024	Rectum, Wound, Nose, Groin, Urine	Türkiye	Türkiye	Yes: I	No
NL-23	I	2024	Bloodˣ, Urine, Throat, Nose, Perineum, Axilla, Groin	Greece	Greece	Yes: I	No
NL-24	III	2024	Axilla, Groin, Wound	Ukraine	Ukraine	Yes: I	No
NL-25	I	2025	Bronchial secretion, Throat, Rectum, Axilla, Wound, Decubitus coccyx, Groin, Catheter, Skin	Saudi Arabia	Saudi Arabia	Yes: I	No
NL-26	I	2025	Urine˚	NA	NA	Yes: S & I	Yes

*N/A = *Not Applicable, *NA = *Not Available. ^º^  = Unknown if other body locations tested positive. ^*^ = Asylum Seeker. ^ˣ^ = invasive infection. *S = *Single−person room with contact precautions, *I* = Isolation room

To assess the risk of nosocomial transmission, isolation measures and contact tracing were evaluated (Table 1). Transmission was not reported by any of the healthcare facilities. Of note, patient NL-07 and NL-13 were not hospitalized in the Netherlands but instead received outpatient care. All 24 remaining patients were admitted in isolation because of their recent travel history, and MDRO screening was conducted. The type of isolation varied between patients. 16 patients were isolated in an isolation room with an anteroom and pressure hierarchy for the entirety of their hospitalization, while 2 patients were exclusively isolated in a regular single person room with contact precautions. 6 patients stayed in both an isolation room and a regular single person room with contact precautions at different times during their hospitalization. All patients, except one, remained in isolation throughout hospitalization because of positive cultures for *C. auris* and/or other MDROs. Only patient NL-04 spent part of their hospitalization not in isolation. This patient was initially admitted in isolation for MDRO screening but was later released from isolation after negative screening tests. After having spent three months in the hospital, the patient tested positive for *C. auris* and was subsequently placed back into isolation in an isolation room.

Contact tracing was performed for patients NL-03, NL-04, NL-10, NL-11, NL-13, NL-17, NL-21 and NL-26. Around patient NL-03, ten internal and two external patients were tested for *C. auris*, but no additional positive cases were identified. Contact tracing was also conducted around patient NL-04, although the exact number of individuals screened is unknown. No transmission was detected. Patient NL-10 was initially admitted to a Dutch hospital following a prolonged stay in a Greek hospital. Afterwards, the patient was transferred to the rehabilitation unit of a separate facility in the Netherlands. Due to the patient’s travel history, MDRO screening was conducted at the Dutch hospital, which revealed the presence of multidrug-resistant *Acinetobacter baumannii.* Consequently, the patient remained in an isolation room throughout the duration of the stay at both the hospital and the rehabilitation unit. After the patient’s transfer to the rehabilitation unit, the patient tested positive for *C. auris*. Contact tracing at the rehabilitation unit for five patients was negative*.* Patient NL-11 was placed in an isolation room at admission but was later moved to contact precautions. After one day in contact precautions the patient tested positive for *C. auris* and was moved back to an isolation room with negative pressure. For contact tracing, a total of 24 individuals were screened for *C. auris,* which were all negative. Patient NL-13 was an asylum seeker, not admitted to a hospital. Two contacts were screened for *C. auris* of which one was found to be *C. auris* qPCR positive. Cultures were negative and thereby genotyping could not be performed, leaving transmission unproven. Patient NL-17 was initially admitted to an isolation room but was later transferred to a single-person room under contact precautions. Upon testing positive for *C. auris*, the patient was moved back to an isolation room. Following the first use of the isolation room by patient NL-17, no complete terminal disinfection was conducted. Consequently, the next patient who occupied the isolation room after patient NL-17 was considered a contact patient and screened for *C. auris*. However, this patient tested negative. Next, patient NL-21 resided in an isolation room throughout hospitalization. Contact tracing involved screening of two patients from the same ward at the time of admission, in addition to screening ICU patients over a two-month period. Lastly, contact tracing was performed for patient NL-26. This patient was residing in a single-person room with contact precautions when they tested positive for *C. auris*. Five patients were screened and all tested negative. None of the individuals screened during contact tracing for all aforementioned patients were found culture positive for *C. auris.*

### Genetic Relatedness

**Fig. 3:**
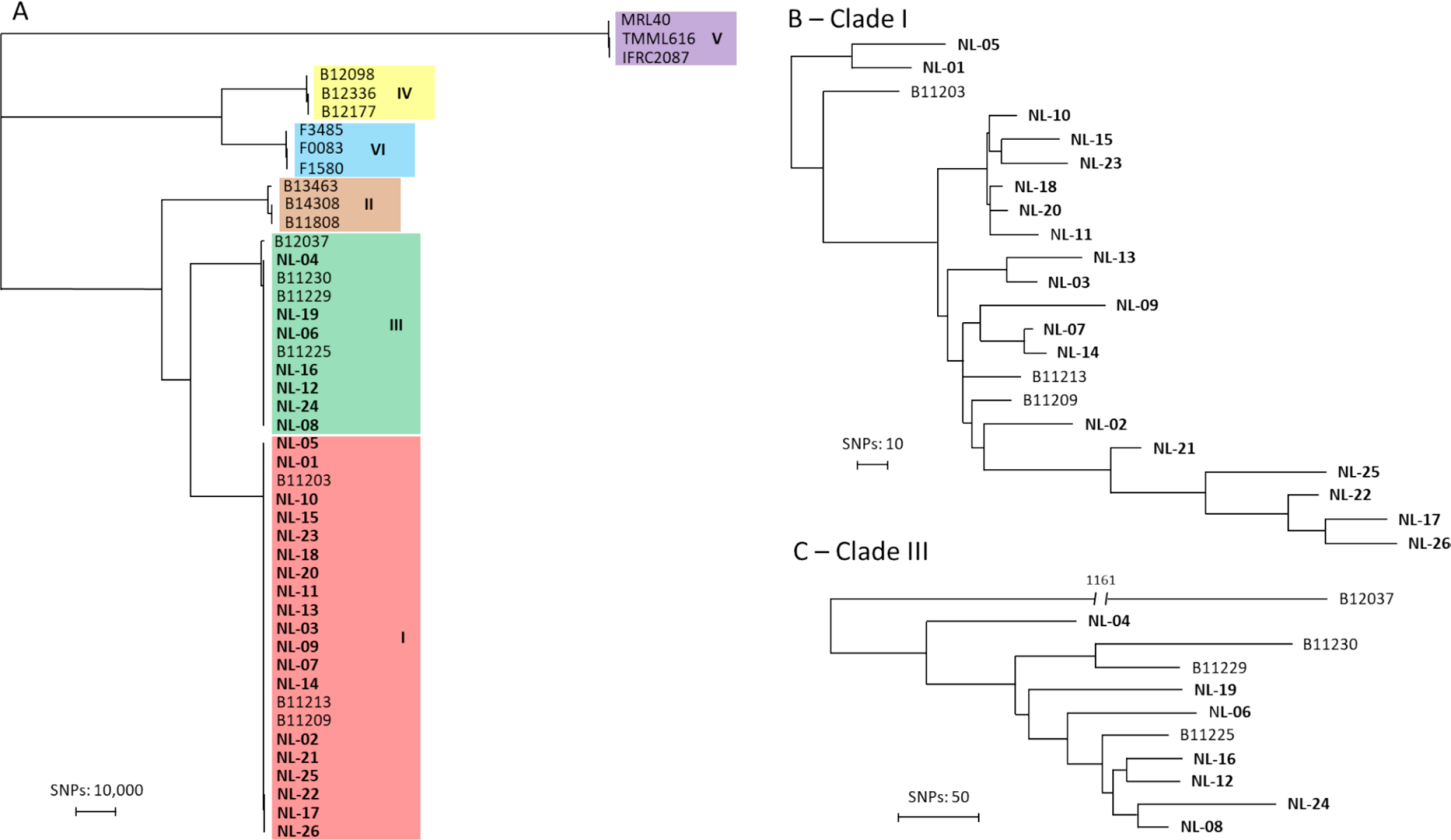
Whole genome sequencing (WGS) single nucleotide polymorphism (SNP) analysis of 26 *C. auris* isolates from the Netherlands (in bold) with controls. **A** All Dutch *C. auris* isolates with controls from the 6 *C. auris* clades. **B** Dutch clade I isolates with controls from clade I. **C** Dutch Clade III isolates with controls from clade III

Initial genotypic screening was conducted on all isolates with STR genotyping. This allocated 19 isolates to clade I, and 7 isolates to clade III (Figure S1). Given that clusters of identical STR genotypes were identified within both clades, whole-genome sequencing (WGS) SNP analysis was performed on all isolates. This analysis subsequently confirmed the clade assignment for all isolates (Fig. 3). For the 19 clade I isolates, a genetic difference of less than 200 SNPs between each other was found. The lowest difference of 8 SNPs was found between isolates NL-07 and NL-14 which originated from patients admitted to different healthcare centers and were collected two years apart. Notably, both patients reported travel history to Tanzania. Additionally, isolates NL-18 and NL-20 were closely related with 9 SNPs difference but again originated from different centers. Both patients had recently been admitted to a Greek hospital. For the seven clade III isolates, a maximum difference of 310 SNPs was found, with all isolates except one (NL-04) forming a monophyletic branch. The minimal SNP difference was 68 SNPs within this clade.

### Resistance

In vitro AFST was conducted according to EUCAST broth microdilution guidelines (Table 2). All fluconazole MICs were elevated, while other azoles were mostly below defined cut-offs [30–32]. MICs for amphotericin B were all wild type. One isolate was non-wild type for 5FC. Interestingly, one isolate was non-wild type for echinocandins and also displayed high MICs against rezafungin, but not ibrexafungerp. MICs for ibrexafungerp and manogepix did not exceed 0.5 mg/L and 0.016 mg/L, respectively. All clade III isolates displayed a fluconazole MIC of ≥ 64 mg/L and harbored the *ERG11*^*VF125AL*^ mutation. Except for two isolates, all remaining clade I isolates harbored the *ERG11*^*Y132F*^ substitution and MICs for fluconazole ranged from 8 mg/L to ≥ 64 mg/L. Of note were two non-wild type isolates that displayed no mutation in *ERG11.* The isolate with reduced susceptibility to echinocandins harbored the *FKS1*^*F635Y*^ mutation.

**Table 2:** Antifungal susceptibility testing and antifungal resistance markers

Non-wild type cutoffs	AMB	5FC	FLC	ITC	VRC	POS	ISA	AFG	MFG	RZF	IBX	MGX	FKS1	ERG11
	> 2	> 0.5	> 4	> 1	> 4	> 0.25	> 0.25	> 0.25	> 0.25	> 0.125	NA	NA		
NL-01	1	< 0.063	** > 64**	0.5	0.25	0.125	**1**	0.031	0.063	0.016	0.25	0.016	WT	WT
NL-02	0.5	< 0.063	**64**	0.016	0.25	< 0.008	0.016	0.031	0.063	0.063	0.063	0.002	WT	Y132F
NL-03	1	0.063	** > 64**	0.063	1	0.031	0.125	0.031	0.031	0.031	0.125	0.008	WT	Y132F
NL-04	1	< 0.063	** > 64**	0.031	0.25	0.008	0.016	0.031	0.063	0.031	0.25	0.004	WT	VF125AL
NL-05	0.5	0.063	** > 64**	0.063	0.5	0.031	0.125	0.063	0.031	0.063	0.5	0.008	WT	WT
NL-06	0.5	0.125	** > 64**	0.016	0.25	0.008	0.016	0.031	0.031	0.031	0.25	0.004	WT	VF125AL
NL-07	1	0.125	**64**	0.031	0.25	0.016	0.016	0.031	0.063	0.016	0.125	0.004	WT	Y132F
NL-08	0.5	0.063	**> 64**	0.031	0.5	0.016	0.031	0.016	0.063	0.031	0.125	0.004	WT	VF125AL
NL-09	2	**128**	** > 64**	1	**8**	0.125	**1**	0.125	0.25	0.031	0.063	0.008	WT	Y132F
NL-10	1	0.125	**32**	0.016	0.125	0.008	0.016	0.063	0.031	0.016	0.25	0.002	WT	Y132F
NL-11	2	0.063	** > 64**	0.031	0.5	0.016	0.063	**2**	** > 4**	** > 0.5**	0.5	0.008	F635Y	Y132F
NL-12	0.5	0.125	**64**	0.031	0.5	0.008	0.016	0.031	0.063	0.031	0.5	0.002	WT	VF125AL
NL-13	1	0.063	** > 64**	0.125	1	0.031	0.125	0.125	0.125	0.031	0.031	0.008	WT	Y132F
NL-14	2	0.063	**64**	0.031	0.25	0.016	0.016	0.016	0.031	0.031	0.5	0.008	WT	Y132F
NL-15	2	0.063	** > 64**	0.063	1	0.016	0.063	0.016	0.063	0.063	0.25	0.008	WT	Y132F
NL-16	0.25	0.125	** > 64**	0.016	0.5	0.008	0.016	0.016	0.031	0.016	0.031	0.008	WT	VF125AL
NL-17	1	0.063	**16**	0.008	0.125	0.016	0.008	0.031	0.063	0.063	0.125	0.002	WT	Y132F
NL-18	1	0.063	**8**	0.008	0.063	0.008	0.008	0.031	0.031	0.031	0.25	0.002	WT	Y132F
NL-19	0.25	0.125	** > 64**	0.031	0.5	0.016	0.031	0.016	0.031	0.016	0.063	0.002	WT	VF125AL
NL-20	1	0.125	** > 64**	0.008	0.125	0.008	0.008	0.031	0.063	0.016	0.25	0.002	WT	Y132F
NL-21	1	0.125	** > 64**	0.063	0.5	0.016	0.063	0.063	0.063	0.031	0.125	0.008	WT	Y132F
NL-22	1	0.125	**16**	0.008	0.125	0.008	0.008	0.063	0.125	0.016	0.25	0.002	WT	Y132F
NL-23	1	0.063	**16**	0.016	0.125	0.008	0.008	0.008	0.031	0.016	0.5	0.002	WT	Y132F
NL-24	0.5	0.125	** > 64**	0.063	1	0.031	0.031	0.031	0.063	0.031	0.25	0.002	WT	VF125AL
NL-25	1	0.125	**16**	0.008	0.125	0.008	0.008	0.004	0.031	0.031	0.25	0.002	WT	Y132F
NL-26	0.5	0.125	** > 64**	0.031	0.5	0.031	0.016	0.016	0.031	0.008	0.25	0.002	WT	Y132F

Non wild type cutoffs were based on published *C. auris* tentative ECV [31] and established EUCAST WT−UL [30]. For fluconazole, the EUCAST non species related clinical breakpoint of 4 mg/L was used [32]. Values that are non−wild type are indicated in bold. WT = Wild type, AMB = Amphotericin B, 5FC = Flucytosine, FLC = Fluconazole, ITC = Itraconazole, VRC = Voriconazole, POS = Posaconazole, ISA = Isavuconazole, AFG = Anidulafungin, MFG = Micafungin, RZF = Rezafungin, IBX = Ibrexafungerp, MGX = Manogepix

## Discussion

Here we provided a comprehensive overview of all *C. auris* cases reported in the Netherlands to date. A total of 26 cases were identified through culture-based methods from the first appearance of *C. auris* in the Netherlands in 2018 up to April 2025. Initially, only one or two cases were reported annually, but since 2023 this number is increasing. Thus far, two cases reported in the Netherlands were invasive infections, the remaining twenty-four cases were regarded as colonization. Analysis of travel history, contact tracing and the WGS data showed no evidence of *C. auris* nosocomial transmission in the Netherlands. All patients, except one, remained in isolation throughout their hospitalization. Isolates showed overall reduced susceptibility to fluconazole and one case of elevated MICs against echinocandins, including rezafungin.

All cases with available travel history, had recently travelled internationally and all but one had also been recently admitted to a foreign hospital. Frequent travel destinations were Greece, India, Ukraine, Tanzania, Türkiye and South-Africa. Especially in Greece, India, Türkiye and South Africa *C. auris* is known to have a high prevalence, while reports from Tanzania and Ukraine are limited, likely due to resource limitations [15, 20, 35]. This emphasizes the importance of screening patients with a recent travel history and/or foreign hospital admission. Since October 2024, *C. auris* is included in the guideline for MDROs by the SRI [23]). This guideline recommends screening of patients who have recently stayed in foreign healthcare facilities, and the isolation of all hospitalized patients colonized with *C. auris*. These measures include placement in a single-bed isolation room with an anteroom and adequate ventilation for source isolation [22]. The guideline further recommends that healthcare workers wear gowns, gloves and masks when entering the room, while also highlighting the critical need for room cleaning and disinfection in addition to standard precautions.

By performing STR genotyping, multiple clusters were found, which could not exclude clonal transmission. However, with WGS SNP analysis, 22 of the 26 isolates had a considerable genetic difference [34, 36]. There were two pairs of isolates with high genetic relatedness, exhibiting 8 and 9 SNP differences, respectively. However, transmission within the Netherlands is unlikely for the first pair due to the prolonged period between sample collections in different hospitals and the shared travel history of both patients to Tanzania. It is likely that clonal transmission occurred involving a clone dominant in Tanzanian hospitals, which both patients independently brought back to the Netherlands. For the second pair, the cases were identified within only a two-month gap, though in different hospitals. However, transmission within the Netherlands is excluded since both patients were directly transferred in isolation from a Greek hospital to a Dutch hospital. Clonal transmission has likely occurred during their hospital stay in Greece, after which they both independently brought back *C. auris* to the Netherlands. It is unknown whether the patients admitted to both the Tanzanian and Greek hospitals had stayed in the same foreign hospital, or in different hospitals within the same country. In regard to all other cases, as far as known, all patients had recently travelled internationally, and there was no epidemiological link between them. Furthermore, all but one patient were kept in isolation throughout their hospital stay. This makes clonal transmission within the Netherlands unlikely, despite the increasing prevalence.

The implemented infection prevention measures and isolation strategies seem to have successfully prevented any transmission, highlighting the importance of isolating *C. auris* patients. Nevertheless, a questionnaire conducted prior to the inclusion of *C. auris* in the MDRO guidelines revealed that many Dutch hospitals were not adequately prepared to handle *C. auris* cases [23, 24]. The findings revealed significant gaps in protocols and variations in practices related to screening, isolation measures, outbreak management, detection and identification. It is therefore possible that transmission may have occurred in the Netherlands without being detected. To prevent unnoticed transmission and future outbreaks, it is crucial for hospitals to establish and uphold robust protocols on screening, detection and infection prevention for *C. auris*, as recommended by the SRI guidelines. Additionally, further research is necessary to address existing knowledge gaps. For example, there is ongoing debate about which body sites should be included in the screening process and whether qPCR-positive cases can transmit the yeast when culture remains negative [19]. In the current study, groin, axilla, urine, wound, and rectum were the most frequently identified culture positive sites. However, this study provides only an overview of the positive screening body sites per patient, rather than a complete list of all screening sites.

In the present study only culture positive *C. auris* cases confirmed by MALDI-TOF MS were included. However, molecular identification using qPCR is increasingly adopted due to its superior sensitivity [26, 27, 37, 38], a practice also being implemented in some Dutch hospitals [24]. In a recent study, 199 patients in a Dutch hospital with a high risk of infection or colonization with an MDRO were screened for *C. auris* using qPCR [26]. While seven patients tested positive for *C. auris* using qPCR, only one of them had a positive culture. Swabs from nose, throat, rectum, axilla, and groin were included, indicating a higher sensitivity for qPCR. However, a large-scale clinical study comparing the sensitivity and selectivity of culture-based methods with qPCR has not yet been performed. In addition to the cases found by Leonhard et al. [26], the authors were aware of several other instances in the Netherlands where qPCR testing was positive, but despite extensive sampling, the cultures remained negative. This included a second patient that resided in a Dutch asylum seeker center. Since only culture positive cases were included in this article, the actual number of *C. auris* patients in the Netherlands is probably higher than presented here when also including qPCR positive cases. Additionally, *C. auris* is currently not a reportable disease in the Netherlands, as this measure is considered too stringent [39]. Laboratories are instead requested to voluntary submit positive cultures to the reference center, which provides useful information on occurrence. Since submission is not mandatory, the reported number of cases in this study could be underreported.

Although no evidence for nosocomial transmission of *C. auris* was observed in the Netherlands to date, the growing number of cases and the distribution of cases across 22 clinical centers, highlights the critical importance of national surveillance. This will be essential for monitoring the incidence of *C. auris*, detecting transmission, and identifying (multicenter) outbreaks. In order to have a complete overview of the incidence of *C. auris,* a registration system should be in place. Other countries, such as England and parts of Belgium, have mandatory reporting for *C. auris* facilitating having such an overview. Currently, *C. auris* is not a reportable disease in the Netherlands, instead laboratories are requested voluntary submit positive cultures to the reference center. However, this approach does not provide insight into qPCR-positive cases. While qPCR may offer high sensitivity, positive cultures are of greater value for surveillance purposes. Isolates can be used to monitor antifungal susceptibility and to confirm or rule out clonal transmission using WGS. On its own, WGS cannot determine whether cases are part of an active outbreak, as strong genetic similarity may result from earlier clonal transmission in other settings. However, when combined with epidemiological data, WGS enables more accurate interpretation of transmission dynamics and supports effective identification and control of potential outbreaks. In addition to the aforementioned more common surveillance methods, wastewater surveillance is gaining interest. Wastewater surveillance has the potential to detect *C. auris* at a low prevalence [40], allowing outbreaks to be identified at an early stage. However, the use of wastewater surveillance for *C. auris* remains in its infancy, and further research is needed to address current knowledge gaps [41].

Finally, the antifungal susceptibility of the 26 isolates in this study showed a pattern in accordance to previous reports for clade I and clade III [42]. Species-specific clinical breakpoints have not yet been established for *C. auris*, which complicates the interpretation of MIC values. Fluconazole MICs were generally high with MICs ≥ 64 mg/L [15]. For clade III the azole resistance-conferring mutation *ERG11*^*VF125AL*^ was found and for clade I this was *ERG11*^*Y132F*^ [42]. Interestingly, four isolates had a MIC of either 8 mg/L or 16 mg/L despite the Y132F mutation, which usually increases the MIC by 8 to 16-fold [42]. A previous study from India also showed the presence of this substitution together with low fluconazole MICs [43], suggesting an unknown mechanism that can increase fluconazole susceptibility. Moreover, two isolates displayed no mutation, suggesting an alternative mechanism may be involved such as upregulation of efflux pumps or increased *ERG11* expression, although this remains to be investigated [42]. One clade I isolate was found to be 5FC non-wild type, though we did not investigate known resistance mechanisms. The single isolate with reduced susceptibility to echinocandins harbored a mutation in *FKS1*, namely F635Y. This mutation is reported occasionally in non-wild type isolates and likely increases the MICs to these agents [42].

## Conclusion

In this study, a comprehensive overview of all 26 *C. auris* cases identified in the Netherlands from 2018 up until April 2025 was provided. The number of *C. auris* cases in the Netherlands are steadily increasing, but no nosocomial outbreaks occurred to date. The included cases are imported from abroad, with no evidence of nosocomial transmission in the Dutch setting, likely due to the implementation of effective infection prevention and isolation measures. The identified strains belonged to clades I and III and were frequently non-wild type for fluconazole, with one isolate being non-wild type for echinocandins including rezafungin. As the number of *C. auris* cases continues to rise, the risk of an outbreak remains a growing concern. This underscores the importance of maintaining robust screening, surveillance, and infection prevention measures to both prevent potential outbreaks and enable a rapid, effective response if they occur.

## Supplementary Information

Below is the link to the electronic supplementary material.

Supplementary file1 (DOCX 198 kb)

## Data Availability

Raw WGS data generated in the current study have been deposited to the NCBI Sequence Read Archive (SRA) database under BioProject PRJNA1272057.
