# Supplementary material for: Candidozyma auris in The Netherlands: No Evidence of Nosocomial Transmission Supported by Effective Infection Control Policies
Source: Mycopathologia. 2026 Jan 10;191(1):17. doi: 10.1007/s11046-025-01024-7 (PMC12789240; doi:10.1007/s11046-025-01024-7)
Supplement: Supplementary file 1 — Supplementary file1 (DOCX 198 kb) [file 11046_2025_1024_MOESM1_ESM.docx]

# Supplementary Tables and Figures

**Table S1:** Overview of control isolates included in the single nucleotide polymorphism (SNP) analysis

| **ID** | **Clade** | **Country** | **SRA** |
| --- | --- | --- | --- |
| B11203 | I | India | SRR14252434 |
| B11209 | I | India | SRR3883441 |
| B11213 | I | India | SRR3883444 |
| B11808 | II | Japan | SRR10461263 |
| B13463 | II | Canada | SRR10461159 |
| B14308 | II | USA | SRR10461147 |
| B11225 | III | South Africa | SRR3883457 |
| B11230 | III | South Africa | SRR3883463 |
| B12037 | III | Canada | SRR10461253 |
| B12098 | IV | Panama | SRR10461248 |
| B12177 | IV | Venezuela | SRR10461201 |
| B12336 | IV | Colombia | SRR7140028 |
| IFRC2087 | V | Iran | SRR9007776 |
| MRL40 | V | Iran | SRR18325430 |
| TMML616 | V | Iran | SRR18325431 |
| F0083 | VI | Bangladesh | SRR25455197 |
| F1580 | VI | Bangladesh | SRR25455198 |
| F3485 | VI | Bangladesh | SRR25455199 |

**
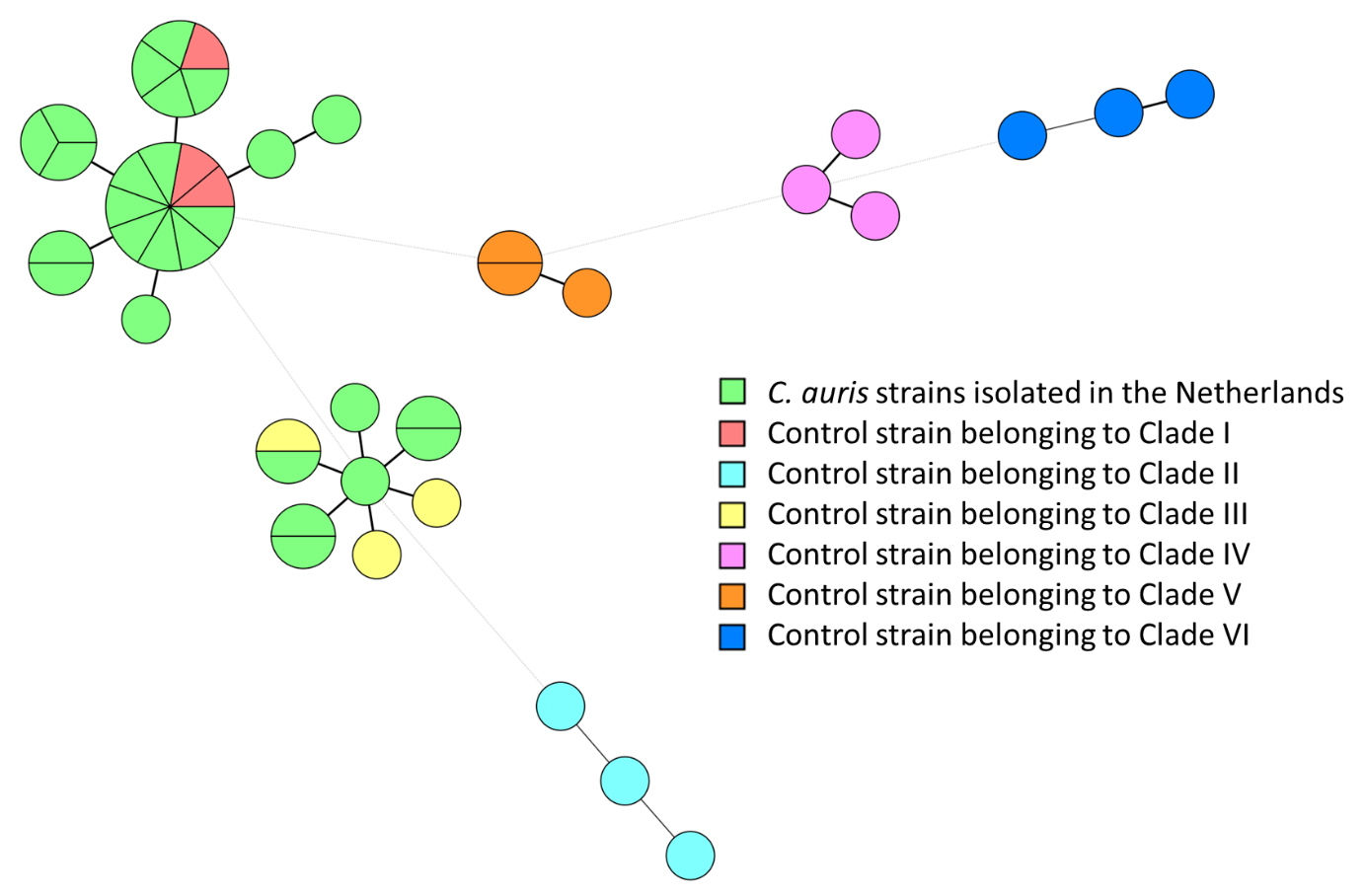
Fig. S1** Minimum-spanning tree from STR typing of 26 Dutch *C. auris* isolates, including control isolates from all six clades. Branch lengths indicate the similarity between isolates based on microsatellite markers, with thick solid lines (variation in one marker), thin solid lines (variation in two markers), thin dashed lines (variation in three markers) and thin dotted lines (variation in four or more markers)
